# A systematic review to investigate the measurement properties of goal attainment scaling, towards use in drug trials

**DOI:** 10.1186/s12874-016-0205-4

**Published:** 2016-08-17

**Authors:** Charlotte M. W. Gaasterland, Marijke C. Jansen-van der Weide, Stephanie S. Weinreich, Johanna H. van der Lee

**Affiliations:** 1 Pediatric clinical Research Office, Academic Medical Center, University of Amsterdam, Meibergdreef 9, 1105 AZ Amsterdam, Netherlands; 2 Department of Clinical Genetics and EMGO Institute for Health and Care Research, VU University Medical Center, BS7, PO Box 7057, 1007 MB Amsterdam, Netherlands

**Keywords:** Rare diseases, Goal attainment scaling, Drug trials, Validation, Systematic review

## Abstract

**Background:**

One of the main challenges for drug evaluation in rare diseases is the often heterogeneous course of these diseases. Traditional outcome measures may not be applicable for all patients, when they are in different stages of their disease. For instance, in Duchenne Muscular Dystrophy, the Six Minute Walk Test is often used to evaluate potential new treatments, whereas this outcome is irrelevant for patients who are already in a wheelchair. A measurement instrument such as Goal Attainment Scaling (GAS) can evaluate the effect of an intervention on an individual basis, and may be able to include patients even when they are in different stages of their disease. It allows patients to set individual goals, together with their treating professional. However, the validity of GAS as a measurement instrument in drug studies has never been systematically reviewed. Therefore, we have performed a systematic review to answer two questions: 1. Has GAS been used as a measurement instrument in drug studies? 2: What is known of the validity, responsiveness and inter- and intra-rater reliability of GAS, particularly in drug trials?

**Methods:**

We set up a sensitive search that yielded 3818 abstracts. After careful screening, data-extraction was executed for 58 selected articles.

**Results:**

Of the 58 selected articles, 38 articles described drug studies where GAS was used as an outcome measure, and 20 articles described measurement properties of GAS in other settings. The results show that validity, responsiveness and reliability of GAS in drug studies have hardly been investigated. The quality of the reporting of validity in studies in which GAS was used to evaluate a non-drug intervention also leaves much room for improvement.

**Conclusions:**

We conclude that there is insufficient information to assess the validity of GAS, due to the poor quality of the validity studies. Therefore, we think that GAS needs further validation in drug studies, especially since GAS can be a potential solution when a small heterogeneous patient group is all there is to test a promising new drug.

**Trial registration:**

The protocol has been registered in the PROSPERO international prospective register for systematic reviews, with registration number CRD42014010619. http://www.crd.york.ac.uk/PROSPERO/display_record.asp?ID=CRD42014010619.

**Electronic supplementary material:**

The online version of this article (doi:10.1186/s12874-016-0205-4) contains supplementary material, which is available to authorized users.

## Background

One of the main challenges for drug evaluation in rare diseases is the heterogeneous course of these diseases. When a disease course differs from patient to patient, traditional outcome measures may not be applicable for all patients of a certain disease. Trial designs are often limited to patients for whom the outcome measure is relevant, whereas the underlying disease mechanism may be similar in a larger group. This increases the problem of small numbers that already challenges rare disease research.

For example, in Duchenne muscular dystrophy (DMD), new drug trials until recently often used the 6-min Walk Test (6MWT) as an outcome measure. The 6MWT has been validated as a reliable and feasible outcome measure, and has been recommended as the primary outcome measure in ambulatory DMD patients [1, 2]. However, although the 6MWT may be a relevant outcome measure for boys who are not (yet) depending on a wheelchair, it is obviously irrelevant for, usually somewhat older, boys who are. This problem in DMD research has been picked up by patient representatives and researchers from all over the world [3].

As the DMD example shows, existing measurement instruments use an outcome that is not relevant for all patients, or may not be responsive enough to measure the effect of an intervention in a rare disease. However, the development of disease-specific and patient-relevant outcome measures is hampered by the small number and heterogeneity of patients with a particular rare disease. In their handbook “Measurement in Medicine” De Vet et al. [4] recommend a minimum number of 50 patients for validation studies.

A measurement instrument that can evaluate the effect of an intervention on an individual basis may help overcome the problem of small, heterogeneous populations. The importance of patient reported outcome measures is widely recognized by pharmaceutical companies and clinical researchers as well as regulators and government agencies such as FDA and NIH [5].

Goal Attainment Scaling (GAS) is a measurement instrument that is intended for individual evaluation of an intervention. It allows patients to set individual goals, together with their treating professional. The number of goals and the content of these goals may differ per patient, but the attainment of the goals is measured in a standardized way. This makes a standardized evaluation of an intervention possible, even when the patients are all in a different stage of their disease.

Goal Attainment Scaling was first introduced in 1968, by Kiresuk and Sherman [6], originally for the evaluation of mental health services. It contains a variable number of self-defined goals and very explicit descriptions of five possible levels of goal attainment that are formulated before the intervention, usually in consultation between the patient and the clinician. In the original definition, the levels are each quantified in a 5-point scale that ranges from −2 to +2, where −2 = the most unfavorable treatment outcome thought likely, −1 = less than expected level of treatment success, 0 = expected level of treatment success, +1 = more than expected success with treatment, and +2 = best conceivable success with treatment. For each goal the expected level of treatment success and at least two other levels need to be described in such a specific way that an independent observer can assess the outcome.

There is no maximum number of goals that can be set. Each goal can be assigned a weight, according to its importance to patient and/or clinician. From the scores reached after the intervention, a composite goal attainment score is computed using the following formula:

$$ T=50+\frac{10{\displaystyle \sum {w}_i{x}_i}}{\sqrt{\left(1-\rho \right){{\displaystyle \sum {w}_i^2+\rho \left({\displaystyle \sum {w}_i}\right)}}^2}} $$

where T is the composite score, w_i_ is the weight assigned to the goal_i_, x_i_ is the original score for goal_i_ ranging from −2 to +2, and ρ is the estimated correlation between goal scores. According to Kiresuk and Sherman, it is safe to assume that the correlation between the goal scores is constant, and can be set at 0.3. The T-score has a mean of 50 and a standard deviation of 10, under the assumptions as proposed by Kiresuk and Sherman [6].

Besides mental health and non-medical fields such as education and social service applications [7], GAS is reportedly used in a few specific medical research areas, such as rehabilitation [8–12] and geriatrics [13–15]. However, the validity of GAS as a measurement instrument in drug studies has never been systematically reviewed. To evaluate the usefulness of GAS in drug studies, we formulated the following three research questions:

1. Has Goal Attainment Scaling been used as a measurement instrument in drug studies?
2. What (drug) interventions were evaluated by studies using GAS?
3. What is known of the validity, responsiveness and inter- and intra-rater reliability of Goal Attainment Scaling in general, and in particular in drug trials?

In this study, we follow the COSMIN guidelines, which are the generally used and accepted standards for measurement properties evaluation [16]. This checklist contains standards for evaluating the methodological quality of studies on the measurement properties of health measurement instruments. According to the COSMIN guidelines, a health status measurement instrument can be used when its validity, reliability and responsiveness, have been tested and considered adequate. We considered GAS useful when the validity, reliability and responsiveness have been described, tested and found acceptable according to these guidelines.

## Methods

We conducted a systematic review, according to the PRISMA guidelines [17].

We set up a sensitive search in Medline, PsychInfo and Embase. We searched for literature from 1968, the year when GAS was introduced by Kiresuk and Sherman [6], to May 1^st^, 2015. For the full search strategy, see Additional file 1. Reference lists of relevant review articles were screened for additional papers.

Papers were included in which:

1. Goal Attainment Scaling met the following criteria:
  One or more individual goals were established by the patient or by one or more researchers or practitioners, either with or without input of the patient, prior to the intervention. The goals did not have to be devised by the patient/researcher, as long as the goals were individually chosen per patient.
  The scale had to consist of at least three points (e.g. more than just goal attained – goal not attained). At least 2 points on the scale were described precisely and objectively, so that an independent observer would be able to determine whether the patient performs above or below that point.
2. The study was either a trial in which drugs are evaluated, or a study of any design in which psychometric properties of GAS were evaluated.
3. The outcome measure was the attainment of goals that had been established before the onset of the intervention.
4. The goals had been set up individually, i.e. per patient.

Excluded were:

1. Trials using an outcome measure called Goal Attainment Scaling, when the outcome measure did not meet our definition of GAS.
2. Studies in which goal setting was used as an intervention rather than outcome measurement.
3. Reviews or narratives.
4. Conference abstracts.
5. Papers published in languages other than English, French, Dutch, German or Spanish.
6. Papers published before 1968.

The selection of articles and data-extraction were performed in pairs of two independent reviewers. Disagreements were discussed until consensus was reached; if necessary a third reviewer acted as a referee. A standardized data-extraction form was used (see Additional file 2). We divided the included studies into two categories, i.e. drug studies, and non-drug studies in which the measurement properties of GAS were investigated.

We extracted information about the following measurement properties, defined according to the COSMIN guidelines [18]: Inter-rater reliability, intra-rater reliability, face validity, content validity, construct validity, and responsiveness. For the full definitions of the measurement properties, see Table 1. We used the quality criteria as proposed by Terwee et al. [19] to evaluate the measurement properties, as also displayed in Table 1. We chose to limit the evaluation of the quality of the measurement properties to the criteria as proposed by Terwee et al., instead of using the full COSMIN guidelines, because the COSMIN guidelines are very detailed, and many details are not relevant as these aspects cannot be evaluated for GAS, e.g. internal consistency, measurement error, criterion validity.

**Table 1:** COSMIN definitions [49] of the evaluated measurement properties, and their quality criteria [19]

Measurement Property	COSMIN definition	Quality criteria (+ equals good to very good quality, +/− equals intermediate quality and – equals poor quality)
Inter-rater reliability	The extent to which scores for patients who have not changed are the same for repeated measurement by different persons on the same occasion	+ ICC^a^ or weighted Kappa ≥0.7 +/− Unclear design or method - ICC or weighted Kappa ≤0.7
Intra-rater reliability	The extent to which scores for patients who have not changed are the same for repeated measurement by the same persons (i.e. raters or responders) on different occasions	+ ICC or weighted Kappa ≥0.7 +/− Unclear design or method - ICC or weighted Kappa ≤0.7
Face validity	The degree to which the items of a Health Related-Patient Reported Outcome (HR-PRO) instrument indeed look as though they are an adequate reflection of the construct to be measured	+ A clear description is provided of the measurement aim, target population, the concepts that are measured, and the item selection and target population were involved in item selection +/− A clear description of these aspects is lacking, or only target population involved, or doubtful design or method - No target population involvement
Content validity	The degree to which the content of an HR-PRO instrument is an adequate reflection of the construct to be measured	+ A clear description is provided of the measurement aim, target population, the concepts that are measured, and the item selection and target population were involved in item selection +/− A clear description of these aspects is lacking, or only target population involved, or doubtful design or method - No target population involvement
Construct validity	The degree to which the scores of an HR-PRO instrument are consistent with hypotheses (for instance with regard to internal relationships, relationships to scores of other instruments, or differences between relevant groups) based on the assumption that the HR-PRO instrument validly measures the construct to be measured	+ Specific hypotheses were formulated and at least 75 % of the results are in accordance with these hypotheses +/− Doubtful design or method (e.g. no hypotheses) - Less than 75 % of hypotheses were confirmed
Responsiveness	The ability of an HR-PRO instrument to detect change over time in the construct to be measured	+ SDC^b^ or SDC ˂ MIC^c^ or MIC outside the LoA^d^ or RR^e^ ˃ 1.96 OR AUC^f^ ≥0.70 +/− Doubtful design or method - Negative SDC or SDC ≥ MIC or MIC equals or inside LOA or RR ≤1.96 OR AUC ˂0.70, despite adequate design and methods

^a^ *ICC* Intraclass Correlation Coefficient

^b^ *SDC* Smallest Detectable Change

^c^ *MIC* Minimal Important Change

^d^ *LoA* Limits of Agreement

^e^ *RR* Responsiveness Ratio

^f^ *AUC* Area Under the receiver operating characteristics Curve

## Results

**Fig. 1:**
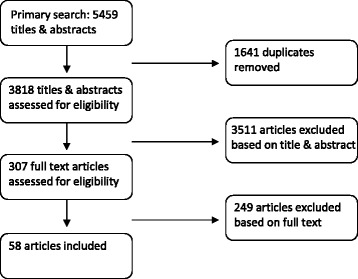
The number of articles in- and excluded in the SR

**Fig. 2:**
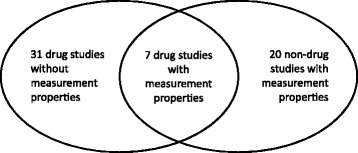
Venn-diagram depicting the number of studies in the categories drug-studies and methodology studies, and the number of studies in both categories

The search yielded 3007, 1413, and 1039 abstracts from Medline, Embase and PsychInfo, respectively. After eliminating duplicates, a total of 3818 abstracts remained for screening. In the screening phase, we excluded 3511 articles based on title and abstract, and 249 articles based on the full text. Data-extraction was executed for the remaining 58 articles (see Fig. 1). Of these 58 articles, 38 articles described drug studies in which GAS was used as an outcome measure, and 20 articles described measurement properties of GAS in other settings (Fig. 2).

In Table 2 the characteristics of the articles are presented. Most studies are trials in patients with cerebral palsy or patients with spasticity due to other causes, such as acquired brain trauma or stroke (28 studies). Also, many studies focussed on the geriatric population (15 studies). There were also some studies on autism (three studies), or neurological disorders such as MS (two studies). The remaining studies covered research areas such as family problems, goal setting in adolescent students or behaviour and psychiatric problems.

**Table 2:** Reported Patients, Interventions, Comparisons and Outcomes in the included studies

Category	First author	Year	Patients	Tested intervention	Comparison	Outcome(s)
Drug study with measurement properties	Cusick [29]	2006	Children with spastic hemiplegic cerebral palsy	Botox-A injections + usual care and occupational therapy	Usual care and occupational therapy	COPM^a^, GAS
Drug study with measurement properties	De Beurs [20]	1993	Patients meeting the DSM-III-R criteria for panic disorder with moderate or severe agoraphobia	Fluvoxamine & exposure in vivo, panic management & exposure in vivo, exposure in vivo only	Placebo with exposure in vivo	GAS, Self-report questionnaires, behavioral avoidance, therapist rating
Drug study with measurement properties	Rockwood [27]	1996	Patients with Alzheimer’s Disease of mild to moderate severity	Linopirdine	placebo	MMSE^b^, ADAS-cog^c^, PSMS^d^, IADL^e^, CGI^f^, GAS
Drug study with measurement properties	Rockwood [50]	2002	Patients with mild to moderate Alzheimer’s Disease	Donepezil hydrochloride 5 mg 1 daily	None	GAS, Cognition (MMSE, ADAS-cog), physical function (PSMS, IADL, FAQ^g^), depression (CDS^h^, CES-D^i^), CIBIC-plus^j^
Drug study with measurement properties	Steenbeek [38]	2005	Children with cerebral palsy	BTX-A treatment of the lower extremity	None	Six-point goal attainment scaling, MAS^k^
Drug study without measurement properties	Ashford [51]	2009	Proximal upper limb spasticity patients	BoNT-A as part of a shoulder and upper limb management and rehabilitation program which was individually tailored to the patient	None	GAS, MAS (composite spasticity score), passive function, shoulder pain
Drug study without measurement properties	Barden [52]	2014-a	Participants with spasticity following acquired brain injury	Botulinum toxin A injections	None	Dynamic Computerized Dynamometry, MAS, Tardieu Scale, Action Research Arm Test, GAS, patient disability and carer burden scales
Drug study without measurement properties	Barden [53]	2014-b	Convenience sample of adults with upper limb spasticity after acquired brain injury with a mean age of 51	BTX-A injections	None	DCD pinch^l^, MAS, Tardieu scale, ARAT^m^, MHOQ^n^, GAS
Drug study without measurement properties	Bonouvrié [54]	2013	Dystonic cerebral palsy patients aged 4–25 years	Continuous intrathecal baclofen for 3 months	Placebo	GAS, measurements of body functions (dystonia, spasticity, pain, comfort, sleep-related breathing disorders)
Drug study without measurement properties	Borg [55]	2011	Adults with a stroke that occurred >3 months before the study	Botulinum toxin A + standard care	Placebo + standard care	GAS, changes from baseline in level of goal achievement, health related Quality of Life, resource utilization
Drug study without measurement properties	Demetrios [56]	2014	Adults with post-stroke spasticity	High intensity ambulatory rehabilitation and Botox	Usual care and Botox	GAS, MAS^o^, participant satisfaction, activity/participation measures and caregiver burden
Drug study without measurement properties	Ferrari [57]	2014	Children with hemiplegic cerebral palsy	BoNT-A injections	Placebo-injections	Body functions and structure, activity and daily life, AHA^p^, MAS, PEDI^q^, GAS
Drug study without measurement properties	Fietzek [58]	2009	Patients with Parkinson camptocormia	Botulinum toxin injections	None	GAS
Drug study without measurement properties	Lam [59]	2012	Patients with severe upper limb spasticity	Intramuscular botulinum toxin A	Saline (placebo)	Carer burden scale, GAS, Ashworth scale, passive range of movement for shoulder abduction, elbow and finger extension, Pain assessment in advanced dementia scale
Drug study without measurement properties	Lam [60]	2015	Long-term care patients with bilateral severe chronic hip adductor spasticity	Ultrasound and electrical stimulation guided obturator nerve block using 5 % phenol	Ultrasound and electrical stimulation guided obturator nerve block using saline	MAS, GAS, hygiene score, distances between the knees, passive range of motion, pain (Pain Assessment in Advanced Dementia Scale), incidence of bone fracture or infections
Drug study without measurement properties	Leroi [61]	2014	Patients with dementia in Parkinson’s disease	20 mg of memantine	Placebo	GAS, Parkinson’s Disease Questionnaire-8, Zarit Burden Inventory
Drug study without measurement properties	Löwe [62]	2006	Children with hemiplegic cerebral palsy, aged 2–8	Occupational therapy & BTX-A injections	Occupational therapy	QUEST^r^, average treatment effect, COPM^s^, GAS, PEDI^t^, Ashworth scale
Drug study without measurement properties	Löwe [63]	2007	Children with hemiplegic cerebral palsy	3 BTX-A injections (0, 6 and 18 months)	2 BTX-A injections (6 and 18 months)	QUEST, GAS-parents, GAS-therapist, COPM, Pediatric Evaluation of Disability, Inventory of functional skills, Ashworth scale
Drug study without measurement properties	Mall [64]	2006	Children with CP and adductor spasticity	BTX-A injections	Placebo	Knee-knee distance, hip adduction, modified Ashworth scale, GMFM^u^, total score and total score without aids, GAS
Drug study without measurement properties	McCrory [65]	2009	Adults with hemiplegic stroke, severe/moderately severe spasticity	Botulinum toxin for upper limbs	Placebo	QoL^v^, GAS, pain, mood, global benefit, MAS^w^, disability and carer burden
Drug study without measurement properties	Molenaers [66]	2013	CP patients with lower limb BTX-A treatment, younger than 24 years of age	BTX-A treatment	None	GAS
Drug study without measurement properties	Nott [67]	2014	Community dwelling adults with acquired brain injury	Botox injections	None	GAS, MAS, TSA^x^, ARAT^y^
Drug study without measurement properties	Olesch [68]	2010	Children with hemiplegic CP	BoNT-A injections + occupational therapy	Occupational therapy alone	COPM^z^, GAS, QUEST^aa^, PDMS-FM^ab^, MTS^ac^
Drug study without measurement properties	Rice [69]	2009	Children with predominantly dystonic CP	Trihexyphenidyl	Placebo	Global dystonia (BAD-scale^ad^), QUEST, COPM, GAS
Drug study without measurement properties	Rockwood [70]	2006	Mild to moderate AD patients	Galantamine	placebo	GAS, ADAS-cog^ae^, CIBIC-plus^af^, DAD^ag^, CBS^ah^
Drug study without measurement properties	Rockwood [71]	2007-a	Patients with mild to moderate AD	Galantamine	placebo	GAS, ADAS-cog, DAD, CBS.
Drug study without measurement properties	Rockwood [72]	2007-b	Patients diagnosed with mild to moderate AD	5 mg of donepezil for 3 months, thereafter flexibly dosed (5 or 10 mg)	None	ADAS-cog, CIBIC-plus, P-GAS, C-GAS
Drug study without measurement properties	Rockwood [73]	2010	Mild to moderate Alzheimer’s Disease patients	Flexibly dosed galantamine for 16 weeks, followed by 16 week open-label phase	Placebo	ADAS-cog, CIBIC-plus, P-GAS and C-GAS
Drug study without measurement properties	Russo [74]	2007	Children (3–16 years) with hemiplegic cerebral palsy	Localized injection of BTX-A and 4 weeks of occupational therapy	4 weeks of occupational therapy	Body structure (Tardieu scale, Ashworth scale), AMPS^ai^, GAS, PEDI^aj^, QoL^ak^
Drug study without measurement properties	Scheinberg [75]	2006	Children aged between 1 and 15 years with CP and clinically significant spasticity	Oral baclofen	Placebo	GAS, MTS^al^, PEDI, parental satisfaction of the effects of the medication
Drug study without measurement properties	Schramm [76]	2014	Patients aged 18 years or older with focal or segmental spasticity showing indication for treatment	Onabotulinum toxin A	None	MAS^am^, spasticity pattern, pain, active hand function, FAC^an^, gait, timed up and go test, goals and treatment parameters, general outcome parameters
Drug study without measurement properties	Turner- Stokes [77]	2007	Patients with regional spasticity following acute stroke or brain injury intervention	Serial injections of botulinum toxin	None	MAS, Associated Reaction rating scale, gait pattern, shoulderQ, functional independence (LASIS^ao^), GAS
Drug study without measurement properties	Wallen [78]	2004	Children with spastic cerebral palsy between the age of 1 and 14 years	Botulinum toxin type A injections	None	COPM^ap^, GAS, Melbourne assessment, CHQ^aq^, parent questionnaire, MAS, Tardieu Scale, range of motion
Drug study without measurement properties	Wallen [79]	2007	Children with CP affecting 1 or both upper limbs, aged 2–14	Single set of BTX-A injections and 12 weeks of occupational therapy	Only occupational therapy or no treatment	COPM, GAS, MAUULF^ar^, CHQ
Drug study without measurement properties	Ward [80]	2009	Children with spasticity and/or dystonia, as classified by a rehabilitation consultant	Intrathecal baclofen therapy	None	COMP^as^, GAS
Drug study without measurement properties	Ward [81]	2014	Adults with focal post-stroke spasticity	Onabotulinumtoxin-A + standard of care	Placebo + standard of care	Number of patients achieving their principal active functional goal, or achieving a different goal at 24 weeks
Non-drug study with measurement properties	Bovend’Eert [37]	2011	Hospital patients with neurological disorders participating in a RCT	A motor imagery program integrated into physiotherapy and occupational therapy; refers to a previous study [82]		
Non-drug study with measurement properties	Brown [32]	1998	Nonambulatory patients who had limited adaptive behavior	Ability-focused physical therapy	None	GAS (treatment goals and control goals)
Non-drug study with measurement properties	Fisher [83]	2002	Patients in a rehabilitation pain management program	Multidisciplinary structured educational program of physiotherapy, occupational therapy and clinical psychology	None	GAS, timed tests of physical mobility measures, MPQ^at^, NRS^au^, ODQ^av^, GHQ^aw^, PAIRS^ax^
Non-drug study with measurement properties	Gordon [30]	1999	Nursing-home patients (elderly and disabled)	Specialized geriatric medicine consultation	None	Effect size and relative efficiency of the Barthel Index, hierarchical assessment of balance and mobility, global deterioration scale, axis 8 (behavior) of the brief cognitive rating scale, cumulative illness rating scale and GAS
Non-drug study with measurement properties	Hartman [39]	1997	Residents of a SCU for persons with dementia	None	None	GAS, COPM^ay^, Cognitive Competency Test, Hierarchic Dementia Scale, Leisure Competence Measure, Leisurescope
Non-drug study with measurement properties	Khan [40]	2008	Persons with MS admitted for comprehensive rehabilitation program	MS rehabilitation program	None	GAS, FIM^az^, Barthel Index, Clinical Global Impression
Non-drug study with measurement properties	Palisano [21]	1993	Infants (4–24 months) with motor delays	2-h intervention session by an interdisciplinary team	None	GAS, Peabody Developmental Gross Motor Scale, behavioral objective, Movement assessment of infants
Non-drug study with measurement properties	Rockwood [31]	1993	Geriatric patients admitted to geriatric inpatient wards	None	None	GAS, Barthel Index, Functional Independence Measure, Physical Self-Maintenance Scale, Katz Activities of Daily Living Index, Spitzer Quality of Life Index
Non-drug study with measurement properties	Rockwood [33]	1997	Patients undergoing cognitive rehabilitation	None	None	GAS, Rappaport Disability Rating Scale, Kohlman Evaluation of Daily Living Skills, Milwaukee Evaluation of Daily Living, Kleinbell elimination scale and mobility scale, Instrumental Activities of Daily Living Scale, Spitzer Quality of Life Index
Non-drug study with measurement properties	Rockwood [41]	2003	Frail elderly	Specialized geriatric intervention	usual care	GAS, Barthel Index, Physical Self-maintenance scale, instrumental activities daily living, modified Spitzer Quality of Life Index
Non-drug study with measurement properties	Ruble [35]	2012	Autism patients	Psychosocial interventions	Unclear	GAS
Non-drug study with measurement properties	Ruble [36]	2013-a	Autism patients	Web based and face-to-face coaching sessions	Placebo	Goal attainment (PET-GAS^ba^), process measures such as consultant and teacher fidelity
Non-drug study with measurement properties	Ruble [34]	2013-b	Autism patients	Face-to-face, Compass intervention/face-to-face, web based compass intervention	Placebo (comparison group)	Child educational outcome, PET-GAS, language ability, autism severity, adaptive behavior, child engagement, maladaptive externalizing behavior
Non-drug study with measurement properties	Sheldon [84]	1998	Undergraduate students	Generating goals	None	A rated attainment scale, GAS
Non-drug study with measurement properties	Steenbeek [44]	2011	Children with cerebral palsy. Aged 2–13 years	Conventional multidisciplinary therapy	None	GAS, PEDI^bb^, GMFM-66^bc^
Non-drug study with measurement properties	Stolee [26]	1999	Geriatric patients	Care as usual	None	GAS, self-rated health, global clinical assessment, Barthel Index, OARS IADL^bd^ scale, MMSE^be^, NHP^bf^
Non-drug study with measurement properties	Stolee [22]	2012	Patients admitted to a geriatric day hospital	Geriatric day program	None	GAS
Non-drug study with measurement properties	Turner-Stokes [42]	2009	Consecutive patients admitted for rehabilitation following acquired brain injury (any cause) over 3 years	Neuro rehabilitation intervention	None	GAS, Functional Assessment Measure (UK FIM + FAM), Barthel Index
Non-drug study with measurement properties	Turner-Stokes [43]	2010	Upper-limb spasticity patients (after stroke)	Intramuscular botulinum toxin-A	Placebo	GAS, MAS^bg^, Global Benefit, HADS^bh^, AQoL^bi^, Patient disability score, Carer burden score
Non-drug study with measurement properties	Turner-Stokes [24, 25]*	2013	Adults with post-stroke upper limb spasticity treated with one cycle of BoNT-A	Botulinum toxin A	None	GAS, spasticity, standardized outcome measures, global benefits
Non-drug study with measurement properties	Woodward [28]	1978	Families with a child between 6 and 16 years of age who was referred for academic or behavioral problems at school	Family therapy	None	GAS
Non-drug study with measurement properties	Yip [23]	1998	Patients admitted to the Geriatric Assessment and Rehabilitation Unit	Rehabilitation interventions	None	GAS (a modified version that uses a standardized menu of goals and attainment levels)

^a^ *COPM* Canadian Occupational Performance Measure

^b^ *MMSE* Mini-Mental State Examination

^c^ *ADAS-cog* Alzheimer’s Disease Assessment Scale - cognitive subscale

^d^ *PSMS* Physical Self-Maintenance Scale

^e^ *IADL* Instrumental Activities of Daily Living

^f^ *CGI* Clinical Global Impression

^g^ *FAQ* Functional Activities Questionnaire

^f^ *CDS* Cardiac Depression Scale

^i^ *CES-D* Center for Epidemiological Studies Depression Scale

^j^ *CIBIC-plus* Clinician’s Interview-Based Impression of Change-Plus

^k^ *MAS* Modified Ashworth Scale

^l^ *DCD* Pinch Dynamic Computerized Dynamometry

^m^ *ARAT* Action Research Arm Test

^n^ *MHOQ* Michigan Hand Outcomes Questionnaire

^o^ *MAS* Modified Ashworth Scale

^p^ *AHA* Assisting Hand Assessment

^q^ *PEDI* Pediatric Evaluation of Disability Inventory

^r^ *QUEST* Quality of Upper Extremity Skills Test

^s^ *COPM* Canadian Occupational Performance Measure

^t^ *PEDI* Pediatric Evaluation of Disability Inventory

^u^ *GMFM* Gross Motor Function Measure

^v^ *QoL* Quality of Life

^w^ *MAS* Modified Ashworth Scale

^x^ *TSA* Tardieu Spasticity Angle

^y^ *ARAT* Action Research Arm Test

^z^ *COPM* Canadian Occupational Performance Measure

^aa^ *QUEST* Quality of Upper Extremity Skills Test

^ab^ *PDMS-FM* Peabody Developmental Motor Scale – Fine Motor

^ac^ *MTS* Modified Tardieu Scale

^ad^ *BAD-scale* Barry-Albright Dystonia scale

^ae^ *ADAS-cog* Alzheimer’s Disease Assessment Scale - cognitive subscale

^af^ *CIBIC-plus* Clinician’s Interview-Based Impression of Change-Plus

^ag^ *DAD* Disability Assessment for Dementia

^ah^ *CBS* Caregiving Burden Scale

^ai^ *AMPS* Assessment of Motor and Process Skills

^aj^ *PEDI* Pediatric Evaluation of Disability Inventory

^ak^ *QoL* Quality of Life

^al^ *MTS* Modified Tardieu Scale

^am^ *MAS* Modified Ashworth Scale

^an^ *FAC* Functional Ambulation Category

^ao^ *LASIS* Leeds Adult Spasticity Impact Scale

^ap^ *COPM* Canadian Occupational Performance Measure

^aq^ *CHQ* Child Health Questionnaire

^ar^ *MAUULF* Melbourne Assessment of Unilateral Upper Limb Function

^as^ *COMP* Canadian Occupational Performance Measure

^at^ *MPQ* McGill Pain Questionnaire

^au^ *NRS* Pain Intensity Numerical Rating Scale

^av^ *ODQ* Oswestry low back pain Disability Questionnaire

^aw^ *GHQ* General Health Questionnaire

^ax^ *PAIRS* Pain and Impairment Relationship Scale

^ay^ *COPM* Canadian Occupational Performance Measure

^az^ *FIM* Functional Independence Measure

^ba^ *PET-GAS* Psychometrically Equivalence Tested Goal Attainment Scaling

^bb^ *PEDI* Pediatric Evaluation of Disability Inventory

^bc^ *GMFM* Gross Motor Function Measure

^bd^ *OARS IADL* Older Americans Resource Scale for Instrumental Activities of Daily Living

^be^ *MMSE* Mini-Mental State Examination

^bf^ *NHP* Nottingham Health Profile

^bg^ *MAS* Modified Ashworth Scale

^bh^ *HADS* Hospital Anxiety and Depression Scale

^bi^ *AQoL* Assessment of Quality of Life

Most drug studies evaluated an intervention with botulinum toxin (25 studies), mainly in patients with cerebral palsy and spasticity. Baclofen was also evaluated in children with spasticity (three studies). Other drugs that were evaluated, were galantamine (three studies), donepezil for Alzheimer’s Disease (two studies), fluvoxamine, trihexyphenidil, memantine, a phenol nerve block, and linopirdine (one study each).

An overview of the reported measurement properties of GAS in the 38 drug studies and the 20 non-drug studies is presented in Tables 3 and 4, respectively.

**Table 3:** Reported measurement properties of GAS in included drug studies

Author	Year	Face validity	Content validity	Construct validity	Intra-rater reliability	Inter-rater reliability	Responsiveness
Cusick	2006	-	-	+	-	-	+
De Beurs	1993	+	-	+	-	+	-
Rockwood	1996	-	-	+	-	-	-
Rockwood	2002	-	-	+	-	-	-
Steenbeek	2005	-	-	-	-	+	-
Turner-Stokes	2010	-	-	+	-	-	+
Turner-Stokes	2013	-	+	+	-	-	-

**Table 4:** Reported measurement properties of GAS in included validity studies

Author	Year	Face validity	Content validity	Construct validity	Intra-rater reliability	Inter-rater reliability	Responsiveness
Bovend’Eert	2011	-	-	-	-	+	-
Brown	1998	-	-	-	-	+	-
Fisher	2002	-	-	+	-	-	-
Gordon	1999	-	-	+	-	-	+
Hartman	1997	-	-	-	-	-	+
Khan	2008	-	-	+	-	-	+
Palisano	1993	-	+	+	-	+	+
Rockwood	1993	-	-	+	-	+	+
Rockwood	1997	-	-	+	-	+	+
Rockwood	2003	-	-	-	-	-	+
Ruble	2012	-	-	-	-	+	-
Ruble	2013-a	-	-	-	-	+	-
Ruble	2013-b	-	-	-	-	+	-
Sheldon	1998	-	-	+	-	-	-
Steenbeek	2011	-	-	+	-	-	+
Stolee	1999	-	+	+	-	+	+
Stolee	2012	-	+	-	-	-	+
Turner-Stokes	2009	-	-	+	-	-	+
Woodward	1978	-	-	+	-	+	-
Yip	1998	-	+	+	-	-	+

### Face validity

As is shown in Tables 3 and 4, face validity is reported in one article [20]. This is a drug study that evaluated the use of Fluvoxamine in patients who met the criteria for panic disorder with moderate to severe agoraphobia. GAS was used as a primary outcome measure. Both therapists and independent raters who assessed the level of goal attainment after the intervention, were asked to rate the relevance of the chosen goals on a scale of 1 to 5 (with one meaning irrelevant and five meaning very relevant). Therapists only rated the GAS score of patients not treated by themselves. The mean score of the therapists was 4.68 (*SD* = .51), and the mean score of the independent raters was 4.66 (*SD* = .52). The researchers concluded that these numbers show that ‘the goal areas were suitably chosen’. The target population of GAS (the patients) were not involved in this evaluation, which is one of the requirements of the quality criteria that we use. However, it is inherent in the measurement instrument that the patient is involved in the choice of the items. Therefore, we score the quality of the face validity evaluation as ‘good quality’.

### Content validity

Content validity was reported in five studies, of which one was a drug study. Content validity was measured in several ways, as shown in Table 5; by rating the usefulness or importance of the goals [21, 22], by comparing the goal areas with essential components as recommended by position papers in the specific field [23] and by checking whether the goals were formulated according to the criteria ‘Specific, Measurable, Assignable, Realistic, and Time-related’(SMART) [24, 25]. In one study, the content validity was reportedly tested by grouping the goals into major categories, and analyzing the content of these categories [26]. However, the study did not report the results of the categorization of the goals [26]. The quality of the content validity varied from ‘good quality’ in two studies, ‘intermediate quality’ in two studies and ‘poor quality’ in one study. Authors reported a ‘good overall usefulness’ of the goals [22], stated that all recommended areas were represented in the goals [23], whether goals were set according to the SMART principle (in this particular study, it was concluded that there was, even after a refinement process of the goal statements, still a difference in the quality of the goal statements between the different sites) [24, 25] or that more than 70 % of the responders rated GAS as a 4 or 5 on a 5-point scale as clinically relevant and important [21].

**Table 5:** Reported content validity of GAS in included studies

First author	Year	Drug study	*N*	Methods and results	Quality
Palisano	1993	No	21	10 physical therapists rated 10 randomly selected GAS-goals on a five-point scale on importance (88 % rated a 4 or 5), the expected level of goal attainment (77 % rated 4 or 5) and clinical relevance (79 % rated a 4 or 5). Between 77 and 88 % of the ratings met the criterion.	+ A clear description is provided of the measurement aim, and target population is inherently involved in item selection
Stolee	1999	No	173	Goals were grouped in major categories, of which the most common were mobility, future care, personal care and bowel and bladder problems. The categorization was reviewed by clinicians of the geriatric rehabilitation unit. The results of this review were not mentioned in the article.	- No results mentioned
Stolee	2012	No	90	Clinicians rated the use of GAS with a mean of 3 (SD 0.9) on a 5-point scale, indicating a “good overall usefulness” of GAS.	+ A clear description is provided of the measurement aim, and target population is inherently involved in item selection
Turner-Stokes	2013	Yes	456	Goal statements for the primary goal in each patient were independently evaluated by three lead clinical investigators, in two rounds. The purpose was to check that clinicians were setting SMART function-related goals in accordance with the training. Goal statements were rated an A, B or C, where an A-rating means ‘Some goal statements contain reference to functional activities at the level of disability or participation—may be ‘active’ or ‘passive’ function’, a B-rating means that ‘Goal statements contain reference to impairment only’, and a C-rating means ‘Goal statements contain reference to anatomical structures only’. Also, a ++, + or – was added, where ++ means ‘There is a SMART goal description, sufficiently detailed and specific to make accurate GAS rating’, + means ‘There is some clear goal description sufficient to support GAS rating, but still reliant on subjective interpretation’ and – means ‘No clear goal \description’. The rating was done in two rounds: after the first round, 62.7 % recorded function-related statements rated A or AB, and 40.3 % of the goal statements received a SMART quality rating of A+/A++. In round two these figures rose to 70.9 and 46.8 % respectively. The authors conclude that even after this goal refinement process, there is residual heterogeneity between the quality of the goals in the different sites that were included in the study.	+ A clear description is provided of the measurement aim, and target population is inherently involved in item selection
Yip	1998	No	143	Content validity was evaluated by comparison of identified goal areas with the essential components of geriatric assessment recommended by several position papers. All the recommended domains were assessed.	+/− Unclear how and by whom the evaluation was scored

### Construct validity

Construct validity was reported in 18 studies, of which six were drug studies (Table 6). In all 18 studies construct validity was assessed by correlations with other instruments measuring a construct similar to the goals that were expected to be set by the patients in each specific research area. Also, T-tests between the placebo and intervention condition [27], or T-tests between the lowest and highest T-score differences [28], were used to verify construct validity. In none of the studies, a hypothesis was formulated on the expected construct validity outcomes. Therefore, the quality of the construct validity is difficult to evaluate. Of the 18 studies, 14 reported significant correlations with other measurement instruments that were relevant for the research area. The measurement instruments used to establish the construct validity varied considerably, since GAS is used for different research areas. Three studies reported that no significant correlations with other measurement instruments were found [21, 29, 30]. In one study correlations between change scores were measured. The results were not clearly reported [31].

**Table 6:** Reported construct validity of GAS in included studies

First author	Year	Drug study	N	Methods and results	Quality
Cusick	2006	Yes	41	Correlations with COMP and GAS Likert scale were measured; no correlation higher than −0.25 or with a *p*-value lower than 0.05.	+/− No hypotheses
De Beurs	1993	Yes	40	Correlations with agoraphobia, rating of treatment outcome by therapist, M-BAT, depression and somatic anxiety were measured; GAS has a high correlation with gain scores on agoraphobia (0.63), rating of treatment outcome by therapist (0.43), and M-BAT (0.57). GAS is moderately correlated with depression (0.32), and not significantly correlated with somatic anxiety.	+/− No hypotheses
Fisher	2002	No	149	Correlations with improvements in walking, general health questionnaire, Oswesty Low Back Pain Disability Questionnaire, NRS and change stand-sit and change PAIRS were measured. There was a significant correlation between GAS and improvements for walking (0.47), between GAS and the general health questionnaire (0.25) and between GAS and the OLBPDQ (−0.31), with *p* <0.01 for all three. No significant correlations were found between GAS and the NRS and change stand-sit and change PAIRS.	+/− No hypotheses
Gordon	1999	No	53	Correlations with standard scales of cognition (MMSE and Global Deterioration Scale), behavior (axis 8 of the brief cognitive rating scale), co-morbidity (cumulative illness rating scale), mobility and balance (hierarchical assessment of balance and mobility, HABAM), and functional capacity (Barthel Index); GAS did not correlate well with any of these measures (correlations varied from −0.22 to 0.17).	+/− No hypotheses
Khan	2008	No	24	Correlation with Barthel Index, Functional Independent Measure and Clinical Global Impression was measured; only the correlation with CGI was significant (−0.77). Also, the difference between responders and non-responders was measured, and a significant difference was found (Z = −3.78, *p* <0.001).	+/− No hypotheses
Palisano	1993	No	21	Correlations between GAS T-scores and Peabody Gross Motor Age equivalent change scores were measured; none of these correlations were significant.	+/− No hypotheses
Rockwood	1993	No	45	Correlations with change scores of Barthel Index, Functional Independent Measure, Mini-Mental State Examination, Katz ADL Index, Physical Self-Maintenance Scale, and Spitzer Quality of Life Index were measured. Correlations varied from −0.87 to 0.84, but it is unclear if these correlations are significant.	+/− No hypotheses, correlations between change scores
Rockwood	1996	Yes	15	A correlation with change scores is measured between GAS and Alzheimer’s Disease Assessment Scale-cognitive, Global Deterioration Scale, Clinical Global Impression, Mini-Mental State Examination, Physical Self Maintenance Scale, and the Instrumental Activities of Daily Living. Correlations varied from −0.85 to 0.74, but it is unclear if these correlations are significant. A T-test between the placebo and the intervention condition was also performed. The T-test showed no difference (*p* = 0.54).	+/− No hypotheses, correlations between change scores
Rockwood	1997	No	44	Correlations with two measurement instruments were measured: Clinical Global Impression (*r* = 0.73) for change score and (*r* = 0.63) at discharge.	+/− No hypotheses
Rockwood	2002	Yes	108	Correlations were measured between several goals within GAS and other measurement instruments. Mini-Mental State Examination and GAS cognition goals: *r* = 0.51. Alzheimer’s Disease Assessment Scale-cognitive and GAS cognition goals: *r* = −0.43. Physical Self Maintenance Scale and clinical function goals: *r* = −0.53. Patient-carer function goals and Physical Self Maintenance Scale: *r* = −0.47. Patient-carer function goals and Instrumental Activities of Daily Living: *r* = −0.44.	+/− No hypotheses
Sheldon	1998	No	82	GAS was correlated with the ‘rated attainment’ scale: *r* = 0.71 (*p* <0.001). There was a correlation with autonomy (*r* = 0.21, *p* <0.01), later effort (*r* = 0.42, *p* <0.01) and autonomous reasons (*r* = 0.09, *p* <0.05).	+/− No hypotheses
Steenbeek	2011	No	23	Correlation with Pediatric Evaluation of Disability Inventory Functional Status Score Mobility: *r* = 0.64 (*p* <0.01), correlation with PEDI Selfcare and social function was not significant.	+/− No hypotheses
Stolee	1999	No	173	Change and follow-up scores of GAS were correlated with Barthel Index, Older Americans Resource Scale Instrumental Activities of Daily Living, Mini-Mental State Examination, Global Rating, Nottingham Health Profile. The correlations varied from −0.31 to 0.67.	+/− No hypotheses
Turner-Stokes	2009	No	164	Correlations were measured between GAS and Functional Independent Measure and Functional Assessment Measure. Correlations with FIM + FAM scores were moderate: 0.36–0.43 for raw scores, 0.41–0.49 for GAS transformed FIM + FAM scores.	+/− No hypotheses
Turner-Stokes	2010	Yes	90	Correlations were measured between GAS and a composite spasticity score (MAS), Global Benefit patient report, Global Benefit investigator report, Hospital Anxiety and Depression Scale anxiety and Hospital Anxiety and Depression Scale depression, Pain at rest, Pain on movement, Assessment of Quality of Life, Patient Disability Score, and Carer burden score. Significant correlations between GAS and MAS (0.35), Global benefit patient report (0.46) and Global benefit investigator-report (0.41) were reported. Other correlations were not significant.	+/− No hypotheses
Turner-Stokes	2013	Yes	456	Correlations between GAS and ‘other measures of outcome, e.g. measures of spasticity, global benefit and other standardized measures’ were calculated. GAS correlated weakly with a reduction in total Modified Ashworth Scale at follow-up (Sp *r* = 0.28, *p* <0.0001) and with global assessment of benefit (*r* = 0.45, *p* <0.0001 for patient assessment, *r* = 0.38, *p* <0.0001 for investigator assessment).	+/− No hypotheses
Woodward	1978	No	279	GAS scores correlate significantly with other outcome measures: *r* = 0.12 - 0.39; *p* <0.05 (in the paper, it is not clear what these other outcome measures are). There was also a difference between the highest and lowest T-score differences: the highest scorers had a mean pre-post score difference of 42.70 (SD = 6.87), the lowest scorers had a mean pre-post difference of 4.05 (SD = 5.78).	+/− No hypotheses
Yip	1998	No	143	Correlations with the Standardized Mini-Mental State Examination, the modified Barthel Index, the Katz Index of ADL and the IADL subscale of the Older Americans Resources and Services Questionnaire were used to demonstrate the convergent construct validity of the standardized menu of GAS. Spearman correlations were calculated between GAS summary scores at discharge and change scores on the Barthel, Katz, OARS-IADL, and SMMSE. The correlations of the total GAS score with changes on the three measures of function were statistically significant but modest (*r* = 0.41 to 0.45); the correlation of GAS with the SMMSE change score was not significant (*r* = 0.11).	+/− Modest correlations

### Intra- and inter-rater reliability

As can be seen in Tables 3 and 4, intra-rater reliability was not assessed in any of the included studies. Inter-rater reliability was reported in 12 studies, of which two were drug studies. Different methods were used to measure the inter-rater reliability (Table 7). In four studies we rated the quality of the inter-rater reliability as poor, whereas eight studies were rated with ‘good quality’. Eight out of the 12 studies reported an ICC score. Five of those studies reported that the ICC values were all 0.9 and higher [31–35]. Two studies reported ICC values between 0.8 and 0.95 [26, 36]. In one study, the reported ICC was lower than 0.5 [37]. The specific calculation for the ICC was reported in one study [37]. Confidence intervals for the ICC values were also reported in one study [35]. Inter-rater reliability was also reported with kappa-values [21, 38], where the values ranged from substantial to almost perfect agreement. Another method that was used was calculating a correlation, which had a value of 0.84 [28]. One study reported ‘agreement’ between objective goal setters and the therapists who performed the interventions, and ‘agreement’ between objective goal setters and people who did the intake of the patients before the patients were randomized. The results were an agreement of 43 and 57 % respectively. However, in the article the method used to calculate this agreement were not reported [20].

**Table 7:** Reported inter-rater reliability of GAS in included studies

First author	Year	Drug study	*N*	Methods and results	Quality
Bovend’Eert	2011	No	29	Mixed model ICC(a, k) between therapist and masked assessor scoring procedures is 0.478 (low); LoA −1.52 +/− 24.54.	- ICC ≤0.7
Brown	1998	No	24	The Pearson’s r correlations and inter-rater ICCs (2,1) between the scores of the treating therapist and the independent raters were *r* = 0.84 (*p* <0.0001, *n* = 360, *r* ^2^ = 70.90/0) and ICC = 1.00 (between raters: (IF = 1, SS = 0.01; within raters: df = 695, SS = 1, 172.65), respectively. The coefficients between scores of the 2 independent raters were *r* = 0.81 (*p* <0.0001, *n* = 135, rZ = 66.2 %) and ICC = 0.997 (between raters: dl = 1, SS = 1.48; within raters: f = 245, SS = 433,39). The results support acceptable inter-rater reliability of the scores for the goals in this study.	+ ICC ≥0.7
De Beurs	1993	Yes	40	Agreement on the content of the chosen goals was measured between the intakers, in other words the people who performed the first session before the patients were randomized, and therapists was measured. Also, the agreement between the therapists and the people who objectively set the goals, or the goal setters, was measured. Agreement between goal setters and therapists and between goal setters and intakers was 43 and 57 % respectively. The calculations used to establish the agreement were not reported.	- Unclear design or method, agreement ≤0.7
Palisano	1993	No	21	Before data collection, an inter rater reliability was measured between the author and an examiner (Kappa = 0.89, agreement 90 %). During the study 16 goals were simultaneously scored. The agreement was 88 % (Kappa = 0.75).	+ ICC ≥0.7
Rockwood	1993	No	45	A primary nurse and a multidisciplinary team scored GAS, ICC = 0.91.	+ ICC ≥0.7
Rockwood	1997	No	44	ICC = 0.95 for admission scoring, ICC = 0.95 for discharge scoring, ICC = 0.93 for change score.	+ ICC ≥0.7
Ruble	2012	No	35 + 44 (reference to previous study)	Two raters independently coded 20 % of the GAS forms for the three features of agreement in sample 1 and 2. ICC for average agreement in sample 1 on measurability (0.96, 95 % CI [.87, .99]), difficulty (0.59, 95 % CI [−.18, .81]) and equidistance (0.96, 95 % CI [.74, .99); ICC for average agreement in sample 2 on measurability (1.0), difficulty (0.96, 95 % CI [.83, .99]) and equidistance (0.96, 95 % CI [.84, .99]).	+ Only ICC for difficulty is lower than 0.7
Ruble	2013-a	No	49	Two coders independently coded 39 % of the goals, ICC for social skills = 0.82, ICC for communication skills = 0.86, ICC for learning skills = 0.91.	+ ICC ≥0.7
Ruble	2013-b	No	Not stated (reference to previous study)	Excellent inter-rater reliability was achieved for both study 1 (ICC = 0.99) and study 2 (ICC = 0.90).	+ ICC ≥0.7
Steenbeek	2005	Yes	11	A video scoring and scoring by a physiotherapist were compared, gaining a Kappa of 0.63. 5 out of 33 of the goal scores differed significantly (tested with a Wilcoxon signed rank test).	- k ≤0.7
Stolee	1999	No	173	ICC (*N* = 61) = 0.93 of GAS follow-up score. ICC (*N* = 61) = 0.89 of the separate goals, when checked whether the goals have been attained.	+ ICC ≥0.7
Woodward	1978	No	279	Correlation of two goal attainment scores: 0.84. 33 % scored identical, 78 % within one level, 95 % within two levels. GAS scores did not differ significantly (F(6,268) = 1.25, *P* >0.10).	- Non-standard way of measuring inter-rater reliability

### Responsiveness

Responsiveness was reported in 14 studies, of which two were drug studies (Table 8). None of the studies used measurement properties as advised by Terwee et al. [19]. Therefore, it is difficult to evaluate the quality of the responsiveness. In nine of those 14 studies, an effect size of the measured differences was reported [26, 29–31, 33, 39–42]. Of those nine studies, the reported effect size was below 1 in only one study [29]. In five studies, a Relative Efficiency was reported [26, 30, 31, 33, 41]. The relative efficiency of two procedures or measurement instruments is the ratio of their efficiencies. For instance, a comparison can be made between GAS and a regularly used measurement instrument. The Relative Efficiency varied between 3 and 57, but was substantial in most studies, meaning that GAS is more efficient, or needs less observations, than other measurement instruments. A Standardized Response Mean was reported in six studies [22, 23, 26, 40–42]. A standardized response mean (SRM) is an effect size index used to measure the responsiveness of scales to clinical change. The SRM is computed by dividing the mean change score by the standard deviation of the change. The SRM’s that were reported varied between 1.2 and 3.54. Two studies measured responsiveness with a paired t-test comparing response before and after the intervention, with a significant difference in GAS T-scores in both studies [22, 39]. In one study, the sensitivity, specificity and positive and negative predictive value were calculated based on a group of responders and non-responders [43]. The results were 52, 85, 81 and 60 %, respectively. In another study, responsiveness was reported as the number of patients who showed a change in T-scores of different goal areas [44]. The proportion of patients showing changes on GAS was larger than on other measurement instruments. The number of patients showing change were nine out of 23 patients on the physical goals, 18 out of 23 patients on occupational goals and 12 out of 18 patients on speech goals, whereas there was only one patient that showed change on the Gross Motor Function Measure (GMFM-66).

**Table 8:** Reported responsiveness of GAS in included studies

First author	Year	Drug study	N	Methods and results	Quality
Cusick	2006	Yes	41	Ability to detect change overtime, and ability to detect difference in change between groups was measured with regression coefficients and effect sizes. Effect size for the weighted GAS scale: 0.55 (*p* = 0.036), and for the Likert scale 0,91 (*p* = 0.003).	+/− Doubtful design or method
Gordon	1999	No	53	GAS was the most responsive measure, with the highest effect size (1.29) and the highest relative efficiency (53.7).	+/− Doubtful design or method
Hartman	1997	No	10	Effect size statistic of 2.34; paired t-test before-after of 2.9 (df = 9, *p* = 0.017).	+/− Doubtful design or method
Khan	2008	No	24	Effect size 9.0, t = 10.0, Standardized response mean = 2.4	+/− Doubtful design or method
Palisano	1993	No	21	Of the 84 goals that were formulated for the study, similar information was obtained with the behavioral objective and GAS formats for 33 (39 %) of the goals, and change that could not be measured with the behavioral objective format was measured with the GAS format for 51 (61 %) of the goals. Of the 17 behavioral objectives that were not achieved, the corresponding GAS score documented progress toward the expected outcome (score of - 1) for 2 (12 %) of the goals. Of the 67 behavioral objectives that were achieved, the corresponding GAS score documented progress that exceeded he criteria for achievement of the behavioral objective (score of +1 or +2) for 49 (73 %) of the goals.	+/− Doubtful design or method
Rockwood	1993	No	45	RE = 4.5; ES = 5.0	+/− Doubtful design or method
Rockwood	1997	No	44	Relative efficiency: 7.8; Effect size: 5.11	+/− Doubtful design or method
Rockwood	2003	No	265	GAS was more responsive than other measures for functional improvement in the elderly; Effect size Cohen’s D: 7.8; SRM: 1.2; NRS: 0.58; Relative efficiency: 57.	+/− Doubtful design or method
Steenbeek	2011	No	23	Individual change score was found in 9/23 (physical), 18/23 (occupational) and 12/18 (speech), and for only one patient a change score was found in the GMFM-66	+/− Doubtful design or method
Stolee	1999	No	173	GAS ES = 3.52; Standardized response mean = 1.73; Relative efficiency = 3.14	+/− Doubtful design or method
Stolee	2012	No	90	All three measures of responsiveness indicated that GAS was able to detect meaningful change in this setting: Paired t-test: T(89) = −17.48; *p* <0.001, SRM = 1.85 (95 % CI 1.50–2.19), ES = 3.27	+/− Doubtful design or method
Turner-Stokes	2009	No	164	SRM: non-weighed GAS = 2.23, weighed GAS = 2.29. Effect sizes: non-weighed GAS = 3.16, weighed GAS = 3.54	+/− Doubtful design or method
Turner-Stokes	2010	Yes	90	The group was divided in responders and non-responders, based on the basis of their mean global benefit at the end of the study; across the whole sample, a change in GAS score from baseline of 6 predicted a positive response, with 52 % sensitivity, 85 % specificity, 81 % positive predictive value and 60 % negative predictive value.	+/− Doubtful design or method
Yip	1998	No	143	Standardized Response Mean was calculated for each instrument, by dividing the mean difference between post-treatment and pre-treatment status by the standard deviation of the mean change score. The SRM was 1.56 for GAS, compared with 0.89, 0.82, 0.72 and 0.54 for the Barthel, Katz, OARS-IADL, and SMMSE, respectively.	+/− Doubtful design or method

## Discussion

In this systematic review, we have found 58 articles, of which 38 drug studies, where GAS was used as an outcome measure. Therefore, we may conclude that GAS has indeed been used in drug studies. Most drug studies that report any information on the validity of GAS, used Botulinum Toxin as an intervention for spasticity, usually in combination with physical or occupational therapy. The generalizability of the results of these validation studies is limited. The validity, responsiveness and reliability of GAS in drug studies have scarcely been studied. In only seven of the 38 drug studies that we found, some validation has been performed. The methods used to validate the measurements instruments often differ from the methods as proposed by COSMIN. The quality of the methods to assess measurement properties varies, and results are often difficult to interpret. We found 20 articles concerning non-drug studies reporting on the validity, responsiveness and inter-rater reliability of GAS. However, also in studies in which GAS was used to evaluate a non-drug intervention, the quality of the validity reports leaves much room for improvement.

In most articles, either drug or non-drug studies, no definition was given of the measurement properties that were assessed, the formulae used for calculation of parameters were not presented, and in some papers the results of the validity check were not reported [26, 31]. Also, none of the included articles describe hypotheses to test construct validity, which makes evaluating the reported results virtually impossible. Therefore, we conclude that the validity and reliability of GAS have not been researched extensively, neither in studies where a drug intervention was evaluated, nor in other studies.

Of all clinimetric characteristics that were investigated, the responsiveness of GAS was investigated most thoroughly. The responsiveness was consistently reported to be very good compared to other measurement instruments, such as the Gross Motor Function Measure (GMFM-66) in the evaluation of children with cerebral palsy, or the Standardized Mini Mental State Examination (SMMSE) for geriatric assessment. However, none of the studies evaluated the responsiveness according to the guidelines as proposed by Terwee et al. [19]. Therefore, it is difficult to be conclusive on the responsiveness of GAS, although the reported results suggest we may tentatively be optimistic.

The search of this systematic review was very sensitive, to make sure that no studies on GAS were missed. However, our definition of GAS is rather specific, which excludes studies with an approach that is similar, but not exactly the same. Also, we may have missed studies that did not use similar terminology, but did use an approach similar to GAS.

Our findings are consistent with previous systematic reviews on the measurement properties of GAS. For instance, Steenbeek et al. [10] concluded that, in the setting of pediatric rehabilitation, GAS is a very responsive method for treatment evaluation and individual goal setting, but sufficient knowledge is lacking about its reliability and validity, particularly. Also, in the field of psychogeriatrics, GAS may be considered useful from a theoretical point of view. Geriatric patients are heterogeneous, and GAS may be a useful tool to evaluate geriatric interventions. However, the measurement properties of GAS in geriatrics show mixed results. The evidence is not yet strong enough to state that GAS is an applicable outcome measure in this particular field [14]. In a systematic review on the feasibility of measurement instruments related to goal setting, GAS is considered a helpful tool for setting goals, although it is time-consuming and may be difficult for patients with cognitive impairments. However, the patient-centered nature of GAS makes it easier to focus on meaningful patient-directed treatment goals. Also, according to the results the scaling of GAS makes it possible to detect very small progress that may be of great significance to the patient, underlining its potential in responsiveness [45].

A problem in the evaluation of the validity of GAS may be that GAS does not measure one clear construct, since the content of the goals generally differs from patient to patient. One of the possibilities to overcome this inherent problem may be to make an item bank of possible goals that patients would be able to choose from, to make sure that the methodological properties of the goals are known [46]. However, this would be practically very difficult to achieve, since we suspect that for many orphan diseases the patient numbers are smaller, and goals could be more diverse than those of non-orphan disease patients. Another way of approaching the construct validity is to see GAS as a measurement instrument that measures the construct of the attainment of goals. Then, the construct validity could be evaluated by comparing GAS with another measurement instrument that evaluates the attainment of goals, such as the COPM. To our knowledge, this approach has not been considered so far.

The importance and difficulty of goals are often taken into account by assigning weights to the goals (more important goals are assigned a larger weight then less important goals). However, terms such as importance and difficulty are by nature subjective. What is important for one patient, may be less important for another. For example, a Duchenne patient may perceive being able to brush his teeth as very important, where someone else may conceive it as trivial. Can this difference in importance objectively be measured? In a study on the reliability of GAS weights, Marson, Wei and Wasserman [47] conclude that assigning weights to the goals of GAS according to the severity of the problem has an acceptable inter-rater reliability when scored by different objective students trained in the use of GAS. This indicates that although importance and difficulty are difficult to objectively measure, objective raters may still score goals similarly. However, more research should be carried out on this topic to answer the question more definitively.

GAS is a measurement instrument with a high potential, especially in rare diseases, but in order to use it in drug studies, more research on its validity is essential. One way of achieving this would be to use GAS as an additional measurement instrument in an ongoing drug trial, to further explore its validity. For GAS to be possibly useful, the effect of the evaluated drug should be objectively measureable in terms of behavior, and it should measure something that is valuable and noticeable for a patient, and cannot be measured otherwise. Also, the drug that is evaluated should have an effect that is also clinically relevant. Again, Duchenne Muscular Dystrophy may serve as an example. A potential drug should do more than just improve for instance the dystrophin values in muscle biopsies. It should be able to improve something that is valuable for the patient, which can be measured by activities that patients perceive as important, such as brushing teeth or using a computer. GAS may be a useful outcome measure, since it can evaluate a potential drug on a patient level, and is therefore intrinsically clinically relevant.

According to guidelines on Patient Reported Outcomes and Health Related Quality of Life by the FDA and EMA, and open comments on these guidelines by experts [48], the following qualities were essential: a PRO should be based on a clearly defined framework, patients should be involved in the development of the measurement instrument, PRO claims should be based on and supported by improvement in all domains of a specific disease, an appropriate recall period is necessary when the effects of an intervention are tested, the test-retest reliability should be assessed, as well as the ability to detect change and the interpretability of the measurement instrument. Finally, an effect found by a PRO measurement instrument can only be valid when found in an RCT.

In general these requirements also apply to GAS, e.g. patient involvement. However, not all of them are applicable to this instrument, such as test-retest reliability. Before GAS can be used in drug trials, more validity research is needed. GAS has not yet been sufficiently validated to be supported by the regulatory agencies, but it may have potential in specific drug trials, especially in rare diseases where there is a lack of validated and responsive outcome measurement instruments.

## Conclusion

We conclude that currently there is insufficient information to assess the validity of GAS, due to the poor quality of the validity studies. However, the overall reported good responsiveness of GAS suggests that it may be a valuable measurement instrument. GAS is an outcome measure that is inherently relevant for patients, making it a valuable tool for research in heterogeneous and small samples. Therefore, we think that GAS needs further validation in drug studies, especially since GAS can be a potential solution when only a small heterogeneous patient group is available to test a promising new drug.

## Abbreviations

ADAS-cog, Alzheimer’s disease assessment scale – cognitive subscale; AHA, assisting hand assessment; AMPS, assessment of motor and process scales; AQoL, assessment of quality of life; ARAT, action research arm test; AUC, Area under the receiver operating characteristics curve; BAD-scale, Barry-Albright Dystonia scale; CBS, Caregiving Burden Scale; CDS, Cardiac depression scale; CES-D, Center for epidemiological studies depression scale; CGI, clinical global impression; CHQ, child heath questionnaire; CIBIC-plus, Clinician’s interview based impression of change-plus; COPM, Canadian occupational performance measure; DAD, disability assessment for dementia; DCD Pinch, dynamic computerized dynamometry; FAC, functional ambulation category; FAQ, functional activities questionnaire; FIM, functional independence measure; GAS, goal attainment scaling; GHQ, general health questionnaire; GMFM, gross motor function measure; HADS, hospital anxiety and depression scale; IADL, instrumental activities of daily living; ICC, intraclass correlation coefficient; LASIS, leeds adult spasticity impact scale; LoA, limits of agreement; MAS, modified Ashworth scale; MAUULF, Melbourne assessment of unilateral upper limb function; MHOQ, Michigan hand outcomes questionnaire; MIC, minimal important change; MMSE, mini-mental state examination; MPQ, McGill pain questionnaire; MTS, Modified Tardieu Scale; NHP, Nottingham health profile; NRS, pain intensity numerical rating scale; OARS IADL, Older Americans resource scale for instrumental activities of daily living; ODQ, Oswestry low back pain disability questionnaire; PAIRS, pain and impairment relationship scale; PDMS-FM, peabody developmental motor scale – fine motor; PEDI, pediatric evaluation of disability inventory; PET-GAS, psychometrically equivalence tested goal attainment scaling; PSMS, physical self-maintenance scale; QoL, quality of life; QUEST, quality of upper extremity skills test; RR, responsiveness ratio; SDC, smallest detectable change; TSA, Tardieu Spasticity Angle

## Additional files

Additional file 1: GAS search. This additional file is the complete search with all the terms that we used to come to the set of articles that we included. (PDF 354 kb) Additional file 2: Data extraction form GAS. This additional file is the complete data extraction form that we have used for the included articles. (PDF 158 kb)
