# Supplementary material for: A systematic review to investigate the measurement properties of goal attainment scaling, towards use in drug trials
Source: BMC Med Res Methodol. 2016 Aug 17;16:99. doi: 10.1186/s12874-016-0205-4 (PMC4989379; doi:10.1186/s12874-016-0205-4)
Supplement: Additional file 1: — GAS search. This additional file is the complete search with all the terms that we used to come to the set of articles that we included. (PDF 354 kb) [file 12874_2016_205_MOESM1_ESM.pdf]

GAS search

Medline

Search terms

1	(goal? adj (set or setting or attainment or scale$ or scaling)).ti,ab,kw.
2	Self Care/
3	self-care.ti,ab.
4	self-set.ti,ab.
5	self-assigned.ti,ab.
6	(individual adj3 goal$).ti,ab.
7	goals/
8	goal$.ti,ab.
9	7 or 8
10	2 or 3 or 4 or 5
11	9 and 10
12	1 or 6 or 11
13	randomized controlled trial.pt.
14	controlled clinical trial.pt.
15	randomized.ab.
16	placebo.ab.
17	drug therapy.fs.
18	drug therapy.fs.
19	trial.ab.
20	groups.ab.
21	Epidemiologic studies/
22	exp case control studies/
23	exp cohort studies/
24	Case control.tw.
25	(cohort adj (study or studies)).tw.
26	(cohort adj (study or studies)).tw.
27	(cohort adj (study or studies)).tw.
28	(observational adj (study or studies)).tw.
29	Longitudinal.tw.
30	Retrospective.tw.
31	Cross sectional.tw.
32	Cross-sectional studies/
33	or/13-32
34	12 and 33
35	(individual* adj5 goal$).ti,ab.
36	33 and 35
37	34 or 36

Embase

	Search terms
1	(goal? adj (set or setting or attainment or scale$ or scaling)).ti,ab,kw.
2	exp *self care/
3	self-care.ti,ab.
4	self-set.ti,ab.
5	self-assigned.ti,ab.
6	(individual$ adj5 goal$).ti,ab.
7	exp *motivation/
8	goal$.ti,ab.
9	7 or 8
10	2 or 3 or 4 or 5
11	9 and 10
12	1 or 6 or 11
13	crossover procedure/ or double-blind procedure/ or single-blind procedure/ or randomized controlled trial/ or crossover$.ti,ab,ot. or cross over$.ti,ab,ot. or placebo$.ti,ab,ot. or (doubl$ adj blind$).ti,ab,ot. or allocat$.ti,ab,ot. or random$.ti,ab,ab. or trial$.ti.
14	*Clinical study/
15	*Case control study/
16	*Family study/
17	*Longitudinal study/
18	*Retrospective study/
19	*Prospective study/
20	*Cohort analysis/
21	(Cohort adj (study or studies)).mp.
22	(Case control adj (study or studies)).tw.
23	(follow up adj (study or studies)).tw.
24	(observational adj (study or studies)).tw.
25	(epidemiologic$ adj (study or studies)).tw.
26	(cross sectional adj (study or studies)).tw.
27	or/13-26
28	12 and 27
29	limit 28 to (conference abstract or conference proceeding)
30	28 not 29

PsycINFO

	Search terms
1	(goal? adj (set or setting or attainment or scale$ or scaling)).ti,ab,tm.
2	exp Self Management/
3	self-care.ti,ab.
4	self-set.ti,ab.
5	self-assigned.ti,ab.
6	(individual$ adj5 goal$).ti,ab.
7	exp Goals/
8	goal$.ti,ab.

9	7 or 8
10	2 or 3 or 4 or 5
11	9 and 10
12	1 or 6 or 11
13	((case* adj5 control*) or (case adj3 comparison*) or case-comparison or control group*).ti,ab,id. not "Literature Review".md.
14	((cohort or longitudinal or prospective or retrospective).ti,ab,id. or longitudinal study.md. or prospective study.md. or retrospective study.md.) not "Literature Review".md.
15	(cross section* or "prevalence study").ti,ab,id.
16	clinical trials/ or "treatment outcome clinical trial".md. or ((randomi?ed adj7 trial*) or ((single or doubl* or tripl* or treb*) and (blind* or mask*)) or (controlled adj3 trial*) or (clinical adj2 trial*)).ti,ab,id.
17	13 or 14 or 15 or 16
18	12 and 17
19	limit 18 to peer reviewed journal
