# Supplementary material for: A systematic review to investigate the measurement properties of goal attainment scaling, towards use in drug trials
Source: BMC Med Res Methodol. 2016 Aug 17;16:99. doi: 10.1186/s12874-016-0205-4 (PMC4989379; doi:10.1186/s12874-016-0205-4)
Supplement: Additional file 2: — Data extraction form GAS. This additional file is the complete data extraction form that we have used for the included articles. (PDF 158 kb) [file 12874_2016_205_MOESM2_ESM.pdf]

DATA EXTRACTION FORM GOAL ATTAINMENT SCALING (GAS)

Name reviewer:____ Date:_______

Goal Attainment Scaling definition

We will only include the papers when:

- One or more individual goals have been established by the patient or by one or more researchers or practitioners, either with or without input of the patient, prior to the intervention. The goals do not have to be devised by the patient/researcher, as long as the goals are individually chosen per patient.

- At least 2 points on the scale are described precisely and objectively, so that an unfamiliar observer can determine whether the patient lies above or below that point. Also, the scale has to consist of at least three points (e.g. more than just goal attained – goal not attained).

We will also include papers when:

- Goals have not been weighed

- There was a limit to the amount of goals that could be chosen

- The scale does not go from -2 to +2

- The formula for calculating a summary T-score as described by Kiresuk & Sherman (1968) is not used

I. GENERAL INFORMATION

Reference number:_______

First author:_________________________________

Year:________

II. IS THE INTERVENTION A DRUG? yes / no

If no: continue with question V.

If yes: continue with question III

III. DESCRIPTION OF STUDY

a) What is the goal of the study?

____

b) What is the design of the study?

____

c) PICO:

Patients: ____

Intervention: ____

Control group: ____

Outcome(s):____

____

____

d) Number of participants:

e) If stated in the article, why is GAS used in this population?

____

____

____

f) Is GAS used as a primary outcome measure or as a secondary outcome measure?

Primary outcome / Secondary outcome

IV. PRACTICAL ISSUES

a) How are goals established?

☐ By the patient alone

☐ By the practitioner alone

☐ By the patient & practitioner together

☐ Other: ………

b) Is it clear how many goals are established per patient? yes / no

If yes:

- Mean amount of goals established in the sample..
- Minimum amount of goals..
- Maximum amount of goals..

c) Was there a maximum amount of goals allowed? yes / no

d) What ratings are used? (E.g. -2 to +2)

____

____

____

e) Analysis: Are ratings summed up using the formula as proposed in Kiresuk & Sherman (1968)?

$$T = 50 + \frac{10\sum w_i x_i}{\sqrt{(1-\rho)\sum w_i^2 + \rho(\sum w_i)^2}}$$ yes / no

If not, how is the data summarized?

____

____

____

f) Are goals weighed according to their value for the patient? yes / no

If yes, how?

____

____

____

V. IS THE VALIDITY/REPRODUCABILITY/RESPONSIVENESS REPORTED?

Yes / no

If yes: continue the data extraction form.

If no: you have finished the data extraction.

VI.

a) Is *face* validity assessed (for example: have researchers superficially checked whether GAS is an adequate instrument to measure goal attainment)? yes / no

If yes, how and result?

____

____

b) Is *content* validity assessed (for example: have experts judged whether GAS is an adequate instrument to measure goal attainment)? yes / no

If yes, how and result?

____

____

c) Is *construct* validity assessed (for example: is GAS correlated with other questionnaires)? yes / no

If yes, how and result?

____

____

VII. <u>REPORTING OF REPRODUCIBILITY</u>

Has an *intra-rater* test-retest been done: yes / no

If yes, result: ____

____

Has an *inter-rater* test-retest been done: yes / no

If yes, result: ____

____

VIII. <u>ASSESSMENT OF RESPONSIVENESS</u>

Was responsiveness assessed: yes / no

If yes, method and result:____

____

____

IX. <u>ADDITONAL REMARKS</u>
